# Brain Wasting

**Published:** 1873-01

**Authors:** 


### BRAIN-WASTING.

An interesting and practical course of clinical lectures on mental and cerebral diseases has been delivered by J. Crichton Brown, M.D., Lecturer on Mental Diseases to the Leeds School of Medicine; and in one on brain-wasting, he states that: 1st. Women recover from this disease more frequently and rapidly than men. 2d. The earlier the age at which brain-wasting occurs, the better is the prospect of recovery. 3d. The more decided the paralytic symptoms, the worse is the prospect of recovery. He also says we owe a debt of gratitude to Dr. Radcliffe for pointing out the value of cod-liver oil and the hypophosphites in debilitating, nervous diseases. They supply the essential elements of nerve-nutrition in an easily assimilable form, and are unmistakably beneficial in cases of brain wasting. A tablespoonful of cod-liver oil, and 15 grs. of the hypophosphite of soda, given twice or three times daily, at the onset of such a case, often arrest at once the downward tendency, and induce restoration of mental and muscular power. Sometimes, when these remedies seem ineffectual, does of from 5 to 15 drops of tincture of opium and sulphuric ether, twice a day, expedite their action, besides conferring independent benefits. The opium gives, as it were, a fillip to cerebral nutrition, and thus diffuses a favorable influence through the whole economy. —*British Med. Journal.*

